# Supplementary material for: COVID-19 Pandemic Impacted Food Security and Caused Psychosocial Stress in Selected States of Nigeria
Source: Int J Environ Res Public Health. 2023 Feb 23;20(5):4016. doi: 10.3390/ijerph20054016 (PMC10001712; doi:10.3390/ijerph20054016)
Supplement: Supplementary file 1 [file ijerph-20-04016-s001.zip › ijerph-2186142-supplementary.pdf]

Supplementary Materials

Table S1. Economic and livelihood data and food-related issues for respondents psychosocial and food security-related impacts of COVID-19, Nigeria.

Variable	Category (n)	Mean ± SD (Naira)	Minimum (Naira)	Maximum (Naira)	$CI_{95\%}$ (Naira)
Income level	Low (287)	55,010.45 ± 25,718.55	5,000	100,000	52,022.35 – 57,998.55
	Medium (82)	196,280.49 ± 86,919.04	110,000	400,000	177,182.28 – 215,378.70
	High (16)	740,375.00 ± 274,546.14	500,000	1,600,000	594,079.62 – 886,670.38
Family monthly transport cost	Low (287)	10,579.37 ± 7,643.57	500	50,000	9,689.74 – 11,469.00
	Medium (82)	30,262.20 ± 18,291.40	2000	100,000	26,243.13 – 34,281.26
	High (16)	73,375.00 ± 51,401.52	20,000	200,000	45,985.05 – 100,764.95
**Other non-monetary parameters**		**Number (%)**	**$CI_{95\%}$**	Comments	
Other income sources (406)	Yes	125 (30.79)	26.50 – 35.44		
	No	281 (69.21)	64.56 – 73.50		
Belong to cooperatives (406)	Yes	64 (15.76)	12.54 – 19.63	Other sources of financial assistance were from churches, friends, and relatives, and outside cooperative groups.	
	No	342 (84.24)	80.37 – 87.46		
Have food items that can last for 14 days during the COVID-19 period (407)	Yes	230 (56.51)	51.66 – 61.24		
	No	177 (43.49)	38.76 – 48.34		
Ran out of food during the COVID-19 pandemic (409)	Yes	173 (42.30)	37.60 – 47.14	150 of the 173 (86.71%) households have ran out of food more than one times during the COVID-19 pandemic	
	No	236 (57.70)	52.86 – 62.40		
Received some assistance from the government (407)	Yes	19 (4.67)	3.01 – 7.18	The assistance were in forms of food palliatives, relief packs, financial assistance, and Nigeria Incentive-based Risk Sharing System for Agricultural Lending (NIRSAL)	
	No	388 (95.33)	92.82 – 96.99		

Family monthly transport cost is 19.23% (18.90 – 19.56) of total incomes for low-income earners, 15.42% (15.26 – 15.58) for middle-income and 9.91% (9.84 – 9.98) for the high-income earners. Family monthly feeding cost is 39.89% (37.62 – 42.16) of total incomes for low-income earners, 37.06% (33.42 – 40.69) for middle-income and 37.44% (27.69 – 47.19) for the high-income earners. As of the time of conducting the survey (26 February 2021), 1 US$ = 380.58 Naira. It should be noted that the figures presented here are based on the responses from respondents during the study and may not be representative of the whole population of Nigeria.

Table S2. Psychological stress and self-rated quality of life of respondents per socioeconomic earning category due to impact of COVID-19

Variable (n)	Income category	Not at all (%)	Only a little (%)	Somewhat much (%)	A great deal (%)
It takes a long time to fall asleep	Low (294)	159 (54.08)	77 (26.19)	47 (15.99)	11 (3.74)
	Medium (96)	62 (64.58)	21 (21.88)	9 (9.38)	4 (4.17)
	High (19)	12 (63.16)	4 (21.05)	3 (15.79)	0 (0.00)
Restless sleep	Low (294)	152 (51.70)	77 (26.19)	53 (18.03)	12 (4.08)
	Medium (96)	64 (66.67)	26 (27.08)	3 (3.13)	3 (3.13)
	High (19)	16 (84.21)	2 (10.53)	0 (0.00)	1 (5.26)
Waking too early and not being able to fall asleep again	Low (294)	146 (49.66)	83 (28.23)	42 (14.29)	23 (7.82)
	Medium (96)	54 (56.25)	27 (28.13)	7 (7.29)	8 (8.33)
	High (19)	15 (78.95)	1 (5.26)	1 (5.26)	2 (10.53)
Feeling tired on waking up	Low (294)	168 (57.14)	81 (27.55)	33 (11.22)	12 (4.08)
	Medium (96)	45 (46.88)	40 (41.67)	8 (8.33)	3 (3.13)
	High (19)	16 (84.21)	0 (0.00)	2 (10.53)	1 (5.26)
Chest, stomach or abdominal pain	Low (294)	220 (74.83)	54 (18.37)	17 (5.78)	3 (1.02)
	Medium (96)	81 (84.38)	11 (11.46)	3 (3.13)	1 (1.04)
	High (19)	18 (94.74)	1 (5.26)	0 (0.00)	0 (0.00)
Heart beating quickly or strongly (palpitation) without a reason like exercise	Low (294)	243 (82.65)	36 (12.24)	13 (4.42)	2 (0.68)
	Medium (96)	78 (81.25)	10 (10.42)	5 (5.21)	3 (3.13)
	High (19)	15 (78.95)	3 (15.79)	0 (0.00)	1 (5.26)
Feeling dizzy or like fainting	Low (293)	240 (81.91)	36 (12.29)	16 (5.46)	1 (0.34)
	Medium (95)	86 (90.53)	6 (6.32)	2 (2.11)	1 (1.05)
	High (19)	15 (78.95)	2 (10.53)	2 (10.53)	0 (0.00)
Feeling pressure or tightness in the head or body	Low (292)	215 (73.63)	61 (20.89)	13 (4.45)	3 (1.03)
	Medium (96)	75 (78.13)	16 (16.67)	4 (4.17)	1 (1.04)
	High (19)	16 (84.21)	1 (5.26)	0 (0.00)	2 (10.53)
Breathing difficulties or feeling of not having enough air	Low (292)	243 (83.22)	29 (9.93)	17 (5.82)	3 (1.03)
	Medium (96)	83 (86.65)	8 (8.33)	5 (5.21)	0 (0.00)
	High (19)	16 (84.21)	2 (10.53)	0 (0.00)	1 (5.26)
Feeling tired or lack of energy	Low (294)	190 (64.63)	55 (18.71)	28 (9.52)	21 (7.14)
	Medium (96)	61 (63.54)	26 (27.08)	7 (7.29)	2 (2.08)
	High (19)	16 (84.21)	2 (10.53)	1 (5.26)	0 (0.00)
Irritable	Low (294)	192 (65.31)	73 (24.83)	24 (8.16)	5 (1.70)
	Medium (96)	73 (76.04)	16 (16.67)	7 (7.29)	0 (0.00)
	High (19)	14 (73.70)	4 (21.05)	0 (0.00)	1 (5.26)
Sad or depressed	Low (293)	175 (59.73)	87 (29.69)	21 (7.17)	10 (3.41)
	Medium (96)	65 (67.71)	23 (23.96)	5 (5.21)	3 (3.13)
	High (19)	14 (73.70)	3 (15.79)	2 (10.52)	0 (0.00)
Feeling tensed or 'wound up'	Low (293)	188 (63.95)	56 (19.05)	41 (13.95)	8 (13.95)
	Medium (97)	67 (69.07)	21 (21.65)	7 (7.22)	2 (2.06)
	High (19)	16 (84.21)	2 (10.52)	0 (0.00)	1 (5.26)
Lost interest in most things	Low (293)	201 (68.60)	60 (20.48)	22 (7.51)	10 (3.41)
	Medium (97)	67 (69.07)	21 (21.65)	6 (6.19)	3 (3.09)
	High (19)	16 (84.21)	2 (10.52)	1 (5.26)	0 (0.00)
Attack or panic	Low (294)	232 (78.91)	38 (12.92)	22 (7.48)	2 (0.68)
	Medium (97)	85 (87.63)	8 (8.25)	4 (4.12)	0 (0.00)
	High (19)	18 (94.73)	1 (5.26)	0 (0.00)	0 (0.00)
Perception of having a physical COVID-19 related problem wrongly diagnosed	Low (293)	256 (87.37)	27 (9.21)	9 (3.07)	1 (0.34)
	Medium (97)	88 (90.72)	7 (7.22)	2 (2.06)	0 (0.00)
	High (19)	19 (100.00)	0 (0.00)	0 (0.00)	0 (0.00)
After reading or hearing about COVID-19, feeling of having similar symptoms	Low (293)	251 (85.67)	22 (7.50)	19 (6.48)	1 (0.34)
	Medium (97)	83 (85.57)	10 (10.31)	2 (2.06)	2 (2.06)
	High (19)	18 (94.73)	1 (5.26)	0 (0.00)	0 (0.00)
When I noticed a sensation in your nose, nostrils, trachea or chest, or I coughed, I find it difficult to think of something else	Low (293)	240 (81.91)	31(10.58)	13 (4.44)	9 (3.07)
	Medium (97)	75 (77.32)	13 (13.40)	4 (4.12)	5 (5.15)
	High (19)	15 (78.95)	2 (10.52)	1 (5.26)	1 (5.26)

Of the total 403 respondents, following the advent of COVID-19, a total of 4 (1.0 %) felt awful/terrible, 41 (10.2 %) felt poor, 152 (37.7 %) felt fair, 145 (36.0 %) felt good and 61 (15.1 %) reported to be with excellent feelings.

Appendix 1

Questionnaire Survey on Psychosocial and Food Security Impacts of novel Coronavirus 2019 (COVID-19) in the Federal Republic of Nigeria

This questionnaire is collated and is being conducted as part of an on-going study on the impact of COVID-19 on food and agriculture in the Federal Republic of Nigeria. The questionnaire also seek to identify psychosocial issues associated with COVID-19 PANDEMIC. It is a non-profit/non-commercial research meant for the public good. The privacy of all participants will be strictly ensured and any information provided will be used only for the purpose of this research.

If you are willing to participate in the research, will you kindly sign the form below:

Name: ………………………………………………………………………… Signature: …………………………… Date: ………………

PART I

SECTION A: GENERAL INFORMATION (HOUSEHOLDS)

s/no.		
1.	District	
2.	Describe Location (GPS if available)	
3.	Name & Surname (optional)	
4.	Marital Status *(please tick (* **X** *where appropriate)*	Married [ ]<br>Separated [ ]<br>Divorced [ ]<br>Widowed [ ]<br>Single [ ]<br>Other (specify) ……………………………

5.	How many are you in your household?	1 – 4 [ ]; 5 – 8 [ ]; 9 – 12 [ ]; > 12 [ ]
6.	Age of respondent *(please tick (* **X** *where appropriate)*<br>*Note: Age is in years.*	Below 20 [ ]<br>20-30 [ ]<br>31-40 [ ]<br>41-50 [ ]<br>Above 50 [ ]
7.	Sex of respondent	Male [ ]<br>Female [ ]
8.	What is your job classification?	
9.	Education level	Number of years of school ……
10.	How many hours do you work per week?	
11.	What is your spouse job?	

12. In term of society class based on income, how will you classify yourself/family?

Class: Low [ ]; Middle income [ ]; High-income [ ].

13. What is the average monthly income for the family? ………………………………………………
14. What is your average monthly transport cost for the family? ……………………………………
15. What percentage of your monthly expenditure is for food and beverages? ………………………..
16. Do you have other sources of income? Yes [ ]; No [ ]
17. If yes, what is it? ………………………………………………………………..
18. Do you belong to any cooperative society or organization (vicoba) that you can borrow money from during this period of coronavirus -19 pandemic? Yes [ ]; No [ ], if '**yes**', state it ……………………………..
19. Where else can you get financial assistance in this period of COVID-19 PANDEMIC (i) Bank (ii) cooperative society (iii) relatives, friends and colleagues?

    ……………………………………………………………………………
20. Do you have some food items that can last for about 14 days at home during this period? Yes [ ]; No [ ]
21. Have you ran out of food in this period of COVID-19 PANDEMIC? Yes [ ]; No [ ], If '**yes**', how many times per week? …………………
22. Did the government consider any form of assistance to you during this period? Yes [ ]; No [ ], If '**yes**', what type of assistance? ………………………………………………………………………………….

---00000---

23. Have you ever been hospitalized? Yes [ ]; No [ ]
24. Please list illnesses, surgical operations and other treatments in recent time and dates (maximum of 5):

S/no.	Recent Illness/Surgery	Approximate dates
1.		
2.		
3.		
4.		
5.		

25. Are you allergic to any drug or substance? Yes [ ]; No [ ]; If '**yes**', specify ……………………………
    ………...
26. What medication are you taking at the moment? …………………………………………………………….
27. Do you drink alcohol? Yes [ ]; No [ ]
28. Do you smoke? Yes [ ]; No [ ]
29. Do you take recreational drugs or substance? Yes [ ]; No [ ]
30. Do you drink coffee or tea? Yes [ ]; No [ ]

SECTION B: FOOD SECURITY EXPERIENCE SCALE QUESTION

Food security and hunger indices assessment

31. On a **scale of 1 – 10**, how will you rate the following questions?

	Food Insecurity Experience Scale Question	Strongly disagreed		Disagreed		Neutral		Agreed		Strongly agreed	
1	Q1. You were worried you would not have enough food to eat because of lack of money or other resources? (if "Yes", go to question Q1a)	1	2	3	4	5	6	7	8	9	10
2	Q1a. Was this specifically due to the COVID-19 crisis?	1	2	3	4	5	6	7	8	9	10
3	Q2. Still thinking about the last 30 days, was there a time when you were unable to eat healthy and nutritious food because of a lack of money or other resources? (if "Yes", go to question Q2a)	1	2	3	4	5	6	7	8	9	10
4	Q2a. Was this specifically due to the COVID-19 crisis?	1	2	3	4	5	6	7	8	9	10
5	Q3. You ate only a few kinds of foods because of a lack of money or other resources? (if "Yes", go to question Q3a)	1	2	3	4	5	6	7	8	9	10
6	Q3a. Was this specifically due to the COVID-19 crisis?	1	2	3	4	5	6	7	8	9	10
7	Q4. You had to skip a meal because there was not enough money or other resources to get food? (if "Yes", go to question Q4a)	1	2	3	4	5	6	7	8	9	10
8	Q4a. Was this specifically due to the COVID-19 crisis?	1	2	3	4	5	6	7	8	9	10
9	Q5. Still thinking about the last 30 days, was there a time when you ate less than you thought you should because of a lack of money or other resources? (if "Yes", go to question Q5a)	1	2	3	4	5	6	7	8	9	10
10	Q5a. Was this specifically due to the COVID-19 crisis?	1	2	3	4	5	6	7	8	9	10
11	Q6. Your household ran out of food because of a lack of money or other resources? (if "Yes", go to question Q6a)	1	2	3	4	5	6	7	8	9	10
12	Q6a. Was this specifically due to the COVID-19 crisis?	1	2	3	4	5	6	7	8	9	10

13	Q7. You were hungry but did not eat because there was not enough money or other resources for food? (if “Yes”, go to question Q7a and Q7b)	1	2	3	4	5	6	7	8	9	10
14	Q7a. How often did this happen?	1	2	3	4	5	6	7	8	9	10
15	Q7b. Was this specifically due to the COVID-19 crisis?	1	2	3	4	5	6	7	8	9	10
16	Q8. You went without eating for a whole day because of a lack of money or other resources? (if “Yes”, go to question Q8a and Q8b)	1	2	3	4	5	6	7	8	9	10
17	Q8a. How often did this happen?	1	2	3	4	5	6	7	8	9	10
18	Q8b. Was this specifically due to the COVID-19 crisis?	1	2	3	4	5	6	7	8	9	10
19	Q9a. Have you experienced increased level of general insecurity in the last 30 days?	1	2	3	4	5	6	7	8	9	10
20	Q9b. Was this specifically due to the COVID-19 crisis?	1	2	3	4	5	6	7	8	9	10
21	Q10a. Have you experienced increased level of dependency in the last 30 days? E.g. more people than usual have called to ask for help.	1	2	3	4	5	6	7	8	9	10
22	Q10b. Was this specifically due to the COVID-19 crisis?	1	2	3	4	5	6	7	8	9	10
23	Q11a. Have you experienced increased level of anger and irritation in the last 30 days?	1	2	3	4	5	6	7	8	9	10
24	Q11b. Was this specifically due to the COVID-19 crisis and its associated pressure?	1	2	3	4	5	6	7	8	9	10

Adapted from the Elbehri (2020). FAO's support to mitigate impact of COVID-19 on food and agriculture in Asia and the Pacific (Version 2.0) and the FAO’s Food Insecurity Experience Scale (FIES) [http://www.fao.org/3/a-bl354e.pdf].

SECTION C: PSYCHOSOCIAL INFORMATION

<u>Psychosocial index evaluation</u> on COVID-19

Considering the situation of COVID-19 specifically, from the beginning of this year 2020, have you experienced the:

32. Death of a family member in relation to COVID-19? Yes [ ]; No [ ]
33. Separation from your spouse or long-term relationship? Yes [ ]; No [ ]
34. Recent change of job/loss of job? Yes [ ]; No [ ]
35. Financial difficulties? Yes [ ]; No [ ]
36. Movement or change of location within the same city? Yes [ ]; No [ ]
37. Movement to another city? Yes [ ]; No [ ]
38. Legal problem? Yes [ ]; No [ ]
39. Begin a new relationship? Yes [ ]; No [ ]

……………………………………………STRESS…………………………………………………….

40. **Do you have a job**? (if yes, answer questions 41-43) Yes [ ]; No [ ]
41. Are you satisfied with your job? Yes [ ]; No [ ]
42. Do you feel more under pressure at work? Yes [ ]; No [ ]
43. Do you have more problem with colleagues at work? Yes [ ]; No [ ]

If **'no' to question 40**, answer the following question:

44. Are you retired or still a student? Yes [ ]; No [ ]
45. Do you feel under pressure during the day? Yes [ ]; No [ ]
46. Are you unable to find a job due to COVID-19? Yes [ ]; No [ ]
47. Do you have serious arguments with close relatives? Yes [ ]; No [ ]
48. Do you have serious arguments with other people? Yes [ ]; No [ ]
49. Has any of the close relatives been seriously ill due to COVID-19? Yes [ ]; No [ ]
50. Do you feel tension at home? Yes [ ]; No [ ]
51. Do you live by yourself? Yes [ ]; No [ ]
52. Do you feel lonely? Yes [ ]; No [ ]

………………………………………..WELL-BEING…………………………………………….

53. Do you have anyone whom you can trust and confide in? Yes [ ]; No [ ]
54. Do you get along well with people in this period? Yes [ ]; No [ ]
55. Do you often feel overwhelmed with the demands of everyday life since the beginning of COVID-19? Yes [ ]; No [ ]
56. Do you often feel that you cannot make it? Yes [ ]; No [ ]
57. Do you tend to be influenced by people with strong opinions whether positive or negative? Yes [ ]; No [ ]
58. Do you tend to worry what other people think of you? Yes [ ]; No [ ]

……………………………………PSYCHOLOGICAL DISTRESS………………………………

On this sliding scale of 1 – 10, can you mark ***'X'*** *on how acutely stressed you have felt*:

Self-reported levels of acute stress during the ongoing pandemic of COVID-19, Nigeria

Before the beginning of COVID-19

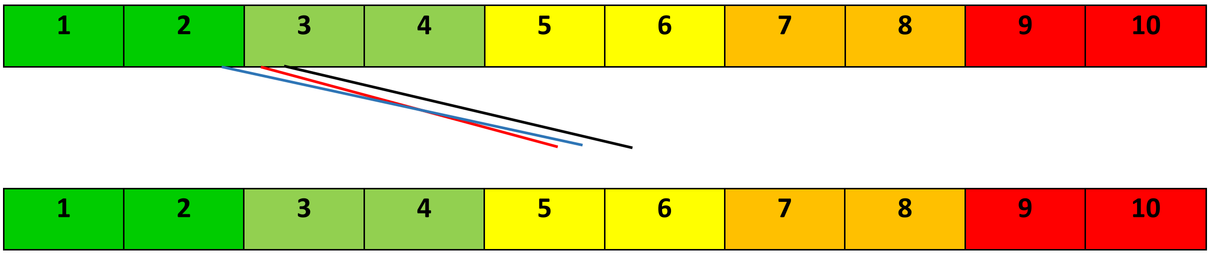

In the ongoing scenario of COVID-19

Note that: 1 = no stress and 10 = much stressed. *(Van Hoof, 2020)*

Black line represents the poor, red line the middle class and blue line the rich. There was significant acute stress observed in each category: Low income earners/poor (n = 289) (slide from 3.28±1.68 to 6.21±2.03, p value < 0.0001); Middle income earners/middle class (n = 87) (slide from 3.18±1.88 to 5.53±2.47, p value < 0.0001); High income earners/upper class (n = 16) (slide from 2.89±1.41 to 5.72±2.52, p value < 0.001);

59. Please describe any problem or difficulties you have had recently in view of COVID-19 and indicate how much these have troubled you by marking the appropriate column.

	**a.** **Not at all:**<br>**b.** **Only a little:**<br>**c.** **Somewhat much:**<br>d. **A great deal:**	a	b	C	d
1.	It takes a long time to fall asleep	☐	☐	☐	☐
2.	Restless sleep	☐	☐	☐	☐
3.	Waking too early and not being able to fall asleep again	☐	☐	☐	☐
4.	Feeling tired on waking up	☐	☐	☐	☐
5.	Chest, stomach or abdominal pain	☐	☐	☐	☐
6.	Heart beating quickly or strongly without a reason like exercise	☐	☐	☐	☐
7.	Feeling dizzy or faint	☐	☐	☐	☐
8.	Feeling pressure or tightness in the head or body	☐	☐	☐	☐
9.	Breathing difficulties or feeling of not having enough air	☐	☐	☐	☐
10.	Feeling tired or lack of energy	☐	☐	☐	☐

a. **Not at all:**<br>b. **Only a little:**<br>c. **Somewhat much:**<br>d. **A great deal:**		a	b	C	d
11.	Irritable	☐	☐	☐	☐
12.	Sad or depressed	☐	☐	☐	☐
13.	Feeling tensed or 'wound up'	☐	☐	☐	☐
14.	Lost interest in most things	☐	☐	☐	☐
15.	Attack or panic	☐	☐	☐	☐
16.	Do you perceive that you have a physical COVID-19 related problem and doctors have not diagnosed it correctly?	☐	☐	☐	☐
17.	When you read or hear about COVID-19, do you feel similar symptoms?	☐	☐	☐	☐
18.	When you notice a sensation in your nose, nostrils, trachea or chest, or you cough, do you find it difficult to think of something else?	☐	☐	☐	☐

Questions 1 – 15 in this section relate to 'Psychological stress' on COVID-19 while 16 – 18 relate to 'Abnormal illness behavior'.

Quality of life

**Mark your answer with X** ☒		Excellent	Good	Fair	Poor	Awful/terrible
1.	How do you rate your overall quality of life since the beginning of COVID-19	☐	☐	☐	☐	☐

This section of the questionnaire is adapted from Piolanti et al., (2016). Use of the psychosocial index: A sensitive tool in research and practice. Psychotherapy & Psychosomatics, 85:337–345

We ask that you think about your life and COVID-19 in the last four months.					
**Scores**	**1**	**2**	**3**	**4**	**5**
	Very poor/very dissatisfied =1	Poor/ dissatisfied = 2	Neither poor nor good/ neither satisfied nor dissatisfied =3	Good/ satisfied =4	Very good/ very satisfied =5
1. How would you rate your quality of life?					
2. How satisfied are you with your health?					
**The following questions ask about how much you have experienced certain things in the last four months related to COVID-19.**					
	Not at all=5	A little=4	A moderate amount=3	Very much=2	An extreme amount=1
3. To what extent do you feel that physical pain prevents you from doing what you need to do?					
4. How much do you need any medical treatment to function in your daily life?					
	Not at all=1	A little=2	A moderate amount=3	Very much=4	An extreme amount=5
5. How much do you enjoy life?					
6. To what extent do you feel your life to be meaningful?					
7. How well are you able to concentrate?					
8. How safe do you feel in your daily life?					
9. How healthy is your physical environment?					
**The following questions ask about how completely you experience or were able to do certain things in the four few months.**					
	Not at all=1	A little=2	Moderately=3	Mostly=4	Completely=5
10. Do you have enough energy for everyday life?					
11. Are you able to accept your bodily appearance?					
12. Have you enough money to meet your needs?					
13. How available to you is the information that you need in your day-to-day life?					
14. To what extent do you have the opportunity for leisure activities?					
	Very poor=1	Poor=2	Neither poor nor good=3	Good=4	Very good=5
15. How well are you able to get around?					

	Very dissatisfied=1	Dissatisfied=2	Neither satisfied nor dissatisfied=3	Satisfied=4	Very satisfied=5
16. How satisfied are you with your sleep?					
18. How satisfied are you with your ability to perform your daily living activities?					
19. How satisfied are you with your capacity for work?					
20. How satisfied are you with yourself?					
21. How satisfied are you with your personal relationships?					
22. How satisfied are you with your sex life?					
23. How satisfied are you with the support you get from your friends?					
24. How satisfied are you with the conditions of your living place?					
25. How satisfied are you with your access to health services?					
**The following question refers to how often you have felt or experienced certain things in the last four months.**					
26. How satisfied are you with your transport?	Never=1	Seldom=2	Quite often=3	Very often=4	Always=5
**Do you have any comments about the assessment?**					

Adapted from WHOQOL

Thank you for your time.
